# Efficient biosynthesis of a Cecropin A-melittin mutant in *Bacillus subtilis* WB700

**DOI:** 10.1038/srep40587

**Published:** 2017-01-10

**Authors:** Shengyue Ji, Weili Li, Abdul Rasheed Baloch, Meng Wang, Hengxin Li, Binyun Cao, Hongfu Zhang

**Affiliations:** 1 Institute of Animal Science, Chinese Academy of Agricultural Sciences, Beijing 100094, China; 2 College of Animal Science and Technology, Northwest A&F University, Yangling, Shaanxi 712100, China; 3 College of Veterinary Medicine, Northwest A&F University, Yangling, Shaanxi 712100, China

## Abstract

The efficient production of antimicrobial peptides (AMPs) for clinical applications has attracted the attention of the scientific community. To develop a novel microbial cell factory for the efficient biosynthesis of a cecropin A-melittin mutant (CAM-W), a recombinant *Bacillus subtilis* WB700 expression system was genetically modified with a novel vector, including a fusion gene encoding CAM-W, the autoprotease EDDIE and the signal peptide SacB under the control of the maltose-inducible promoter P_*glv*_. A total of 159 mg of CAM-W was obtained from 1 L of fermentation supernatant. The purified CAM-W showed a consistent size with the expected molecular weight of 3.2 kDa. Our findings suggest that this novel expression system can be used as a powerful tool for the efficient production of CAM-W.

Antimicrobial peptides (AMPs) have recently become powerful chemotherapeutic alternatives for targeting drug-resistant bacterial pathogens due to their mechanism of action (i.e., outer membrane permeation). Moreover, the use of AMPs may reduce the likelihood of the emergence of bacterial resistance^1^,^2^,^3^,^4^. A four-tryptophan-substitution mutant (KWKL***W***KKIEK***W***GQGIGAVLK***W***LTT***W***L-NH2; CAM-W)^5^ from cecropin A-melittin (KWKLFKKIEKVGQGIGAVLKVLTTGL-NH_2;_ CAM)^6^ has recently been developed through the replacement of particular amino acid residues by four tryptophans (W). CAM-W shows more potent antimicrobial activity against a wide range of clinically important bacterial and fungal pathogens (e.g., *Escherichia coli* and *Aspergillus flavus*) than its parental peptide CAM. Furthermore, CAM-W shows improved proteolytic stability under a series of proteases, including trypsin, pepsin, and the *Staphylococcus aureus* V8 protease, which are commonly present in the gastrointestinal tract (GIT)^5^. The pH range in the GIT is suitable for the function of CAM-W^5^; thus, CAM-W has potential for clinical applications.

The efficient production of CAM-W is required for commercial production and applications. Recombinant DNA technology and chemical synthesis are suitable for CAM-W production because the single polypeptide chain of CAM-W consists of common amino acids^7^. However, because chemical synthesis is cost-intensive, an efficient biosynthetic approach would be better suited for CAM-W production. Several recombinant small peptides have been successfully expressed in *E. coli*^8^,^9^ and *Pichia pastoris*^10^,^11^ expression systems. However, the expression of heterologous proteins in *E. coli* often results in inclusion bodies that do not exhibit any biological activity and requires solubilization, refolding and purification procedures to recover functionally active products^12^. While the use of a yeast expression system such as *P. pastoris* may address the issue of post-translational modifications^10^, such systems require a significant investment during the latter period of batch cultivation, making this approach uneconomical.

*Bacillus subtilis* expression systems possess the capacity to secrete proteins into the extracellular space with their biological activity intact. Additionally, these systems have a short fermentation period due to their rapid growth rate. These systems have been employed for the biosynthesis of several heterologous proteins, while avoiding the inclusion bodies and uneconomical fermentation problems associated with the *E. coli*^13^ and *P. pastoris*^14^ culture processes, respectively^15^. Of the different types of *B. subtilis* host strains, *B. subtilis* WB700 is deficient in seven extracellular proteases. Thus, this bacteria presents the outstanding advantage of minimizing the degradation of secreted proteins within the extracellular space, which in turn increases the production of target proteins^15^.

Several protein fusion technologies have been used to facilitate the expression of heterologous proteins. One such practice is the small ubiquitin-related modifier (SUMO) fusion technology^16^,^17^. SUMO can dramatically enhance expression and chaperone correct protein folding in the soluble form. This fusion technology has recently been used to express several AMPs, including scolopin 1^18^, CM4^19^ and plectasin^20^, in *E. coli* expression system. However, the purification procedure, which includes breaking host cells to release the fusion proteins and supplementing additional SUMO protease to liberate the protein of interest from SUMO, somewhat inhibits the application of this technology.

The N^pro^ autoprotease originates from classical swine fever virus and has an autoproteolytic function. This function allows it to release its fusion partner from the C-terminal end of the autoprotease by self-cleavage, resulting in a target protein with an authentic N-terminus^21^. Furthermore, its tailor-made mutant (EDDIE) shows improved solubility and faster cleavage than its parent and has already been applied to improve heterologous expression by overcoming challenges such as inefficient cleavage and proteolytic degradation^22^. EDDIE has been applied to produce CM4 in host *E. coli* strains and to protect host cells from CM4-induced damage; however, the fusion protein was expressed in inclusion bodies, and a complicated procedure was required to obtain the active product^23^. In the present study, the chimeric protein EDDIE was fused with CAM-W to shield the host *B. subtilis* WB700 strain from CAM-W-induced damage. Similar technology has been used to express AMP buforin II by neutralizing the buforin II positive charges through fusion with an acidic peptide, thereby avoiding the damage associated with the production of buforin II in *E. coli* strains^24^. To the best of our knowledge, this is the first report on the use of EDDIE fusion technology as a bi-functional tool to protect host strains during the CAM-W biosynthesis process and to self-cleave the fusion protein to release CAM-W. Accordingly, a novel *B. subtilis* WB700 expression system based on EDDIE fusion technology was developed to facilitate the efficient production of CAM-W.

## Materials and Methods

### Construction of recombinant strains

**Figure 1:**
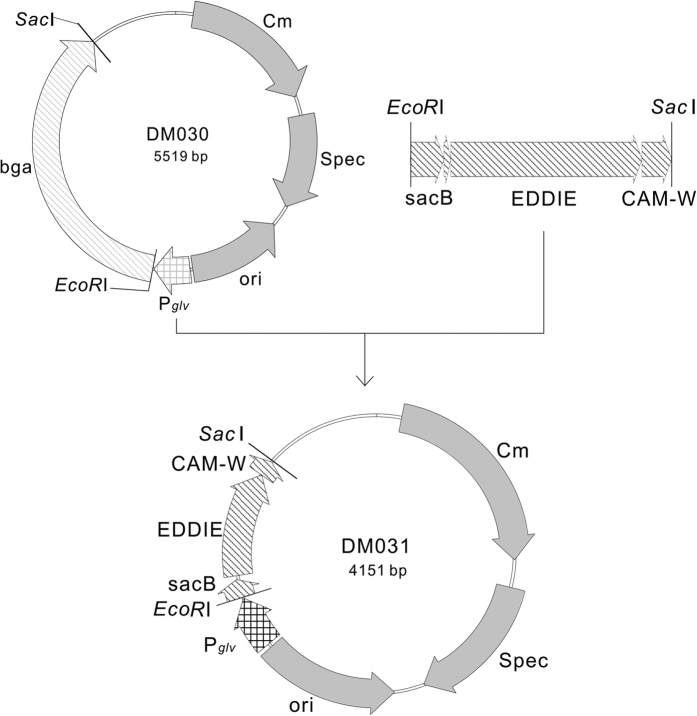
Plasmid construction of pDM031. The recombinant plasmid harbors the inducible promoter P_*glv*_and the fusion gene encoding the signal peptide SacB and EDDIE-CAM-W.

Vector pDM030 (kept in our laboratory) is a shuttle vector that can replicate in both *E. coli* and *B. subtilis* and harbors the promoter P_*glv*_^25^,^26^ and genes for chloramphenicol and spectinomycin resistance and β-galactosidase. The vector was digested with *Eco*RI and *Sac*I. A 702-bp fusion fragment encoding SacB-6His-EDDIE-CAM-W with *Eco*RI and *Sac*I sites was synthesized at AuGCT Co., Ltd. (Beijing, China) and cloned into the vector by displacing the β-galactosidase gene to yield pDM031 (Fig. 1). *E. coli* DH5α was transformed with recombinant pDM031, and positive clones were selected with 5 μg/ml of chloramphenicol. The transformants were verified by sequencing at AuGCT, and the verified pDM031 was transformed into *B. subtilis* WB700 (kindly donated by Dr. Sui-Lam Wong^27^) using an electroporation approach^28^.

### Biosynthesis of CAM-W

Positive clones of recombinant *B. subtilis* WB700 strains were selected from Luria-Bertani (LB) agar containing 50 μg/ml spectinomycin. These clones were cultured in 50 ml of LB broth^26^ in a 250-ml shaker flask at 37 °C with an agitation speed of 225 rpm. At 12 h post-inoculation, when the recombinant strains reached the late logarithmic growth phase, the culture broth was supplemented with maltose solution to a final concentration of 5%^26^ for a subsequent 12-h culture. Then, the supernatants were harvested by centrifugation at 12,000 × *g* for 10 min at 4 °C. The *B. subtilis* WB700 strain was used as a negative control and was treated under parallel conditions.

### Isolation of total RNA and real-time PCR

Cultures were collected every 3 h after the start of fermentation until 36 h. Total bacterial RNA was isolated using an SV total RNA isolation kit (#Z3100; Promega, USA). The extracted total RNA was reverse-transcribed into cDNA using a reverse transcription system kit (#A3500; Promega, USA). Real-time PCR was subsequently performed using a real-time PCR kit (#DRR041S; TaKaRa, Japan). The gene encoding SacB-EDDIE-CAM-W was amplified with primers (eddie-up: GTGGAGGAACCAGTGTATGA and eddie-down: GTCCCATGTAGTCCTGGTAA). The 16 S rDNA of the *B. subtilis* WB700 strain was amplified as a control using the 16s-up/16s-down primers. The PCR protocol was performed as follows: 2 min at 50 °C and 10 min at 95 °C, followed by 35 cycles consisting of 42 s at 95 °C, 60 s at 49 °C, and 30 s at 72 °C. The reactions were performed in an IQ5 real-time PCR detection system (Bio-Rad, USA).

### Western blotting

The crude peptides in the supernatants were mixed with 4 × Laemmli loading buffer (3:1) and heated in boiling water for 5 min. The treated samples were subjected to Tris tricine sodium dodecyl sulfate polyacrylamide gel electrophoresis (tricine-SDS-PAGE)^29^ with a 10% gel and were electrotransferred to a PVDF membrane (Millipore, USA) for protein immunoblot analysis. Mouse anti-EDDIE monoclonal antibody (mAB) and mouse anti-CAM-W mAB were prepared and purified by Cwbiotech (Beijing, China). After incubation with the appropriate HRP-conjugate secondary antibody, the signals were detected using a ChemiDoc XRS imaging system and QuantityOne analysis software (Bio-Rad, USA).

### Purification of CAM-W

The culture broth was centrifuged at 7000 × *g* for 10 min, and the supernatant was filtered through a 0.22-μm pore filter (Nucleopore, Costar). A total of 100 μl of crude peptides was subjected to a reversed phase (RP) high-performance liquid chromatography system (HPLC; Agilent, USA) with a semi-preparative Zorbax 300SB-C8 column (250 mm × 9.4 mm, 5-μm particle size, 300-Å pore size) (Agilent, USA). The column was equilibrated in 0.1% (v/v) trifluoroacetic acid and 10% acetonitrile and then developed with a linear 0% to 60% acetonitrile gradient at a flow rate of 1.0 ml/min. The absorbances at 214 and 280 nm were monitored, and the peaks were investigated using an antimicrobial activity assay with *E. coli* serving as the indicator strain^5^. The purified peptides were verified using an RP-HPLC analytical Zorbax 300SB-C8 column (250 mm × 4.6 mm, 5 μm, 300 Å) (Agilent, USA) and were freeze-dried in a vacuum freeze dryer (SIM International Group Co., Ltd., USA) at −80 °C for further experiments. The concentration of the purified peptide solutions was determined by UV spectrophotometry^30^,^31^. The molecular weight of the purified CAM-W was measured using electrospray ionization mass spectrometry (Agilent, USA).

### Antibacterial activity

The solid phase method was used to chemically synthesize CAM-W (GL Biochem Ltd., Shanghai, China). Five different bacteria, including *E. coli* 15224, *Pseudomonas aeruginosa* ATCC 90271, *S. aureus* ATCC 29213, *Shigella sonnei* ATCC 25931 and *Streptococcus pyogenes* ATCC 10389, were obtained from the American Type Culture Collection (ATCC; Rockville, MD, USA) and used as indicator strains. The antibacterial activities of chemically synthesized and biosynthesized CAM-W against the indicator strains were investigated based on a previously reported microtiter broth dilution method^5^.

### Statistical analysis

Each experiment was repeated three times, and the mean values are expressed as the mean ± standard deviation (SD).

## Results

### Construction of recombinant *B. subtilis* WB700 strains

As shown in Fig. 1, we constructed an inducible expression plasmid (pDM031) that harbored an operon including the maltose-inducible P_*glv*_ promoter and a fusion fragment encoding SacB-EDDIE-CAM-W. Then, recombinant *B. subtilis* WB700 strains capable of expressing EDDIE-CAM-W were constructed by electroporation with pDM031.

### Growth of recombinant strains

**Figure 2:**
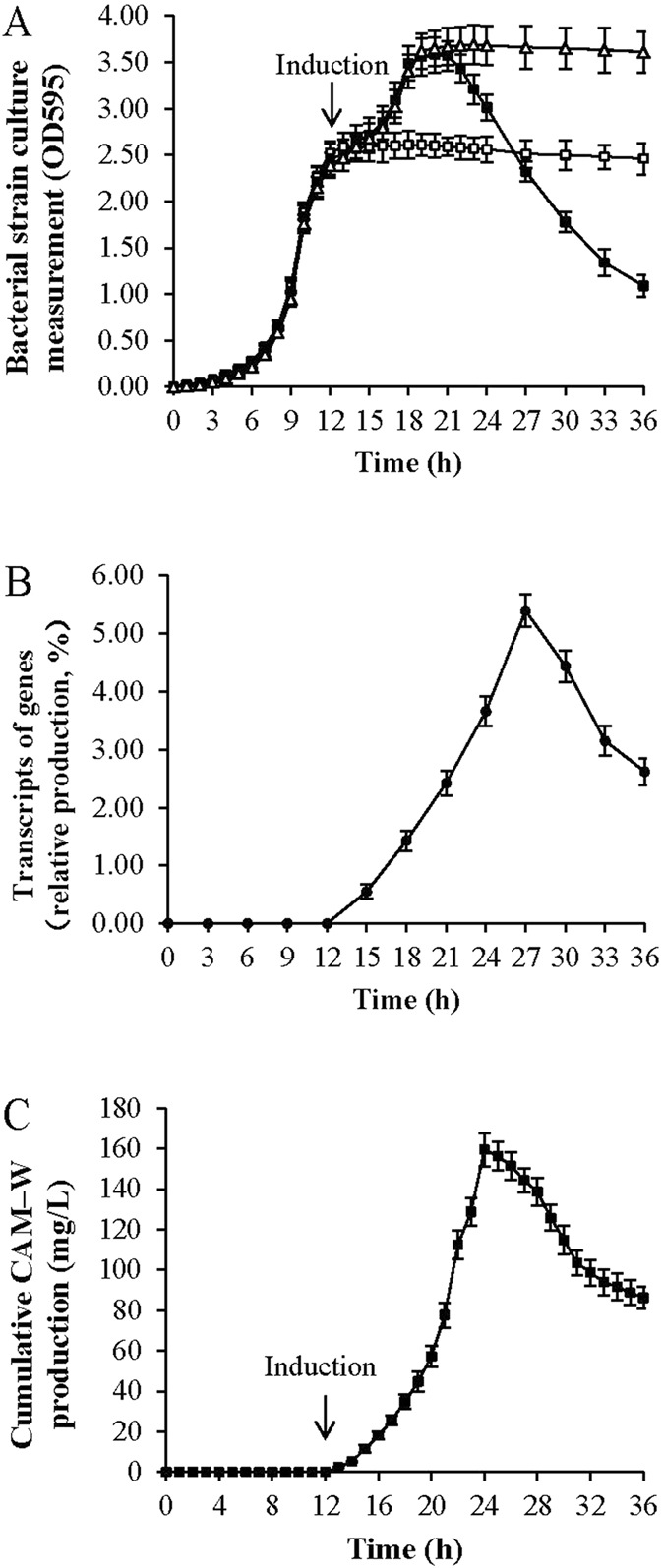
Biosynthesis of CAM-W in *B. subtilis* WB 700 induced by maltose. (**A**) The OD_595_ measurement of *B. subtilis* WB700 harboring or not harboring pDM031. *B. subtilis* WB700 induced by maltose (∆), *B. subtilis* WB700 harboring pDM031 not induced by maltose (□), and *B. subtilis* WB700 harboring pDM031 induced by maltose 12 h after fermentation (■). (**B**) Cumulative CAM-W production in the culture supernatant of *B. subtilis* WB700 harboring pDM031 induced by maltose. The maximum production of 159 mg CAM-W was obtained from 1 L of culture supernatant after 24 h of fermentation. (**C**) Real-time PCR analysis of the transcription of genes encoding SacB-EDDIE-CAM-W from *B. subtilis* WB700 harboring pDM031 following induction by maltose. The 16 S rDNA of *B. subtilis* WB700 was amplified using the 16s-up/16s-down primers and was used as a control.

The *B. subtilis* WB700 strains harboring/not harboring pDM031 reached the late logarithmic growth phase 12 h after the start of culture (Fig. 2A). Thereafter, the strains reached another logarithmic growth phase following the addition of maltose, with maximum OD_595_ values > 3.5. However, following maltose induction during the first late logarithmic growth phase, the amount of recombinant *B. subtilis* WB700 significantly declined during the second late logarithmic growth phase, 18 h after the start of culture, compared with the non-recombinant *B. subtilis* WB700 strain.

### Expression of CAM-W

After maltose induction during the first late logarithmic growth phase (Fig. 2A), the promoter P_*glv*_ efficiently promoted transcription of the fusion gene encoding SacB-EDDIE-CAM-W (Fig. 2B). CAM-W expression became detectable after maltose induction (Fig. 2C) and reached a maximum level of 159.46 mg CAM-W from 1 L of fermentation culture after 24 h of recombinant strain growth. However, the amount of CAM-W began to decline 12 h after maltose induction from the maximum value of 159.46 mg/L, and the level was reduced by nearly half after 36 h of culture in the recombinant strain.

### Identification of CAM-W

**Figure 3:**
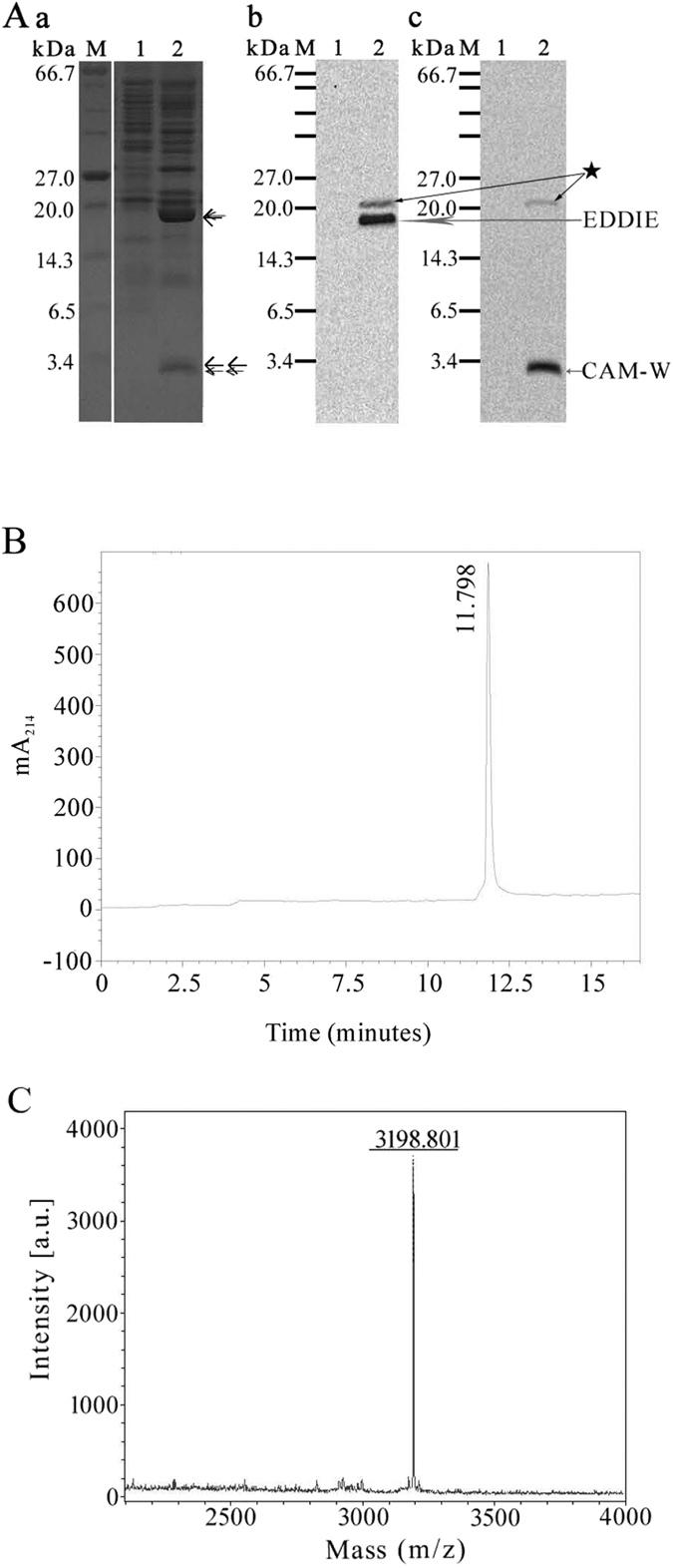
Identification of CAM-W produced by recombinant *B. subtilis* WB 700. (**A**) Analysis of tricine-SDS-PAGE (a) cropped from Supplementary Figure 1 and western blots (b and c) cropped from Supplementary Figures 2 and 3 of the total extracellular proteins from *B. subtilis* WB700 harboring pDM031 induced without maltose (lane 1) or with maltose (lane 2). These gels were run under the same experimental conditions. The EDDIE and CAM-W bands are indicated by (←) and (← ← ). The bands present in (a) lane 2 were confirmed by western blotting, as shown in (b) and (c). The marker lane contained a broad range protein marker (#P7702, New England Biolabs, USA). (**B**) RP-HPLC analysis of purified CAM-W. (**C**) Electrospray ionization mass spectrometry analysis of purified recombinant CAM-W.

Tricine-SDS-PAGE analysis of the total extracellular proteins from the recombinant *B. subtilis* WB700 strains showed that the molecular masses of CAM-W and EDDIE were 3.2 kDa and 19 kDa, respectively (Fig. 3A). Western blot analysis confirmed the results of tricine-SDS-PAGE analysis and demonstrated that CAM-W and EDDIE were present in the culture supernatants (Fig. 3A-b and 3A-c). Purified CAM-W was obtained from the culture supernatants using the RP-HPLC purification process, subsequently verified using the RP-HPLC analytical process with retention time 11.798 min (Fig. 3B), and subjected to further analysis. Electrospray ionization mass spectrometry showed that the molecular mass of CAM-W was 3198.801 Da (Fig. 3C), which is consistent with the predicted value of 3197.9 Da and the detection of a 3.2-kDa protein by tricine-SDS-PAGE analysis (Fig. 3A-a). Furthermore, the results for the biosynthesized CAM-W were similar to those for chemically synthesized CAM-W in terms of antibacterial activity against the five tested bacteria (Table 1).

## Discussion

Currently, the secretory expression of AMPs of interest is important for both basic research and practical applications. Compared with the commonly used *E. coli*^32^,^33^ and yeast^34^,^35^ expression systems, *B. subtilis* has been recognized as an ideal model for studying the secretory expression of proteins of interest in Gram-positive bacteria due to its ability to secrete proteins directly into the medium, short fermentation period, well-characterized genetic background, non-pathogenic status, and broad industrial applications^36^,^37^,^38^. *B. subtilis* WB700 not only has the advantages of expressing and secreting common *B. subtilis* proteins, but is also characterized by the absence of 7 extracellular proteases that partially protect the secreted proteins of interest from degradation^15^. Thus, the *B. subtilis* WB700 strain was selected to produce CAM-W in this study.

In addition to the choice of producer strain, the combination of vector and promoter is also important for the expression of target AMPs (i.e., CM4 based on the pSUMO vector with the T7 promoter produced in the *E. coli* expression system^19^ and hPAB-β based on the pPIC9K vector with the AOX1 promoter biosynthesized in the *P. pastoris* expression system^34^). Nonetheless, the development of an inducible secretory expression system that is dependent on the use of a vector capable of efficient replication with a strong and controllable promoter is much more attractive. Of the numerous vectors that have been constructed and applied in *B. subtilis*, pUB110 has two replication origins that result in an enhanced replication efficiency^39^. Thus, pUB110 was used to develop pGJ203^25^, which is the parental plasmid of the pDM030 and pDM031 vectors used in this study. P_*spac*_^40^, P_*xylA*_^41^, and constitutive promoters such as P43^42^ are commonly used in *B. subtilis* expression systems. However, the expression level of the P_*glv*_-dependent system is significantly higher than that of the P43-dependent system. Moreover, the use of maltose as an inducer is more economical than the use of xylose or IPTG in the P_*xylA*_- or P_*spac*_-dependent systems. It is noteworthy that a new inducible *B. subtilis* secretory expression system based on the pGJ203 vector with the P_*glv*_ promoter was recently developed and showed an outstanding ability to secrete the expressed target proteins^25^,^26^.

The recombinant plasmid pDM031, which is derived from the pGJ203 vector^25^ and carries the maltose-inducible promoter P_*glv*_^26^ and a DNA fragment encoding the signal peptide SacB, was constructed for the efficient biosynthesis of CAM-W (Fig. 1). Then, a recombinant *B. subtilis* WB700 strain harboring pDM031 was developed. P_*glv*_ played a vital role in CAM-W biosynthesis when the producer grew to the first late logarithmic phase (Fig. 2A) by promoting over-transcription following the addition of maltose to the fermentation broth (Fig. 2B)^25^,^43^. Additionally, following the initiation of maltose-induced expression, the signal peptide SacB, *via* the Sec translocation pathway, directed the fusion protein EDDIE-CAM-W from the cytoplasm into the culture medium in an unfolded and inactive form^44^,^45^. This step was important for shielding the host strain from the bactericidal action of active CAM-W (Fig. 2A). Moreover, the producer strain *B. subtilis* WB700 was deficient in seven extracellular proteases^15^ and thus offered a significant advantage of minimizing degradation and improving the production of CAM-W. Based on the distinct outstanding characteristics of these primary elements, the novel recombinant *B. subtilis* WB700 expression system is endowed with potential for biosynthesizing CAM-W (Fig. 2C).

However, in the each western blot the other bands as showed with pentagram were observed (Fig. 3A-b and 3A-c), that indicated uncleavage fusion protein and suggested a not high efficient cleavage rate for lysine as the first amino acid in the sequence of CAM-W, and this finding was consistent with the report that illustrated EDDIE released model peptied K169-VDKLAAALEHHHHHH on *in vitro* refolding with a 56% cleavage rate^21^. In spite of this, this finding demonstrated that the EDDIE expression system could be used as a generic tool for the high-level production of recombinant toxic peptides in *B. subtilis*.

Although the extracellular space of *B. subtilis* WB700 strain is dificient in seven extracellular proteases, where still exists other proteases such as *wpr* (cell-wall-associated protease) that may result in some degradation of target proteins, such as the decreasing trend of penicillin G acylase in previous report^46^ and CAM-W in current work, additionally the released CAM-W may in a certain extent cause damage to the host strains, a high-level production of CAM-W was obtained with the participation of SacB and P_*glv*_ and under the action of the autoprotease EDDIE due to the large amount of producer strains present (Fig. 2A and C). In the current study, the biosynthesized CAM-W achieved a level of up to 159.46 mg/L in the culture supernatant (Figs 2C and 3A). This level was higher than that obtained in *P. pastoris* (125 mg/L^10^). In addition, after purified from the culture supernatants, CAM-W was verified using RP-HPLC analytical process with retention time at 11.798 min (Fig. 3B), and subsequently purified CAM-W showed a molecular weight consistent with the theoretical prediction (Fig. 3C) and exhibited an antibacterial activity similar to that of the chemically synthesized peptide (Table 1). Although the difference was not significant, CAM-W from the *B. subtilis* expression system required no more than 24 h of fermentation time, which is approximately one third of the fermentation time required for CAM-W from the *P. pastoris* expression system. In other words, the productivity of CAM-W from the *B. subtilis* expression system was three times higher than that from *P. pastoris* expression system. Thus, the P_*glv*_-dependent expression system may be more suitable for efficient CAM-W biosynthesis.

In conclusion, an efficient recombinant *B. subtilis* WB700 expression system for CAM-W biosynthesis was developed for the first time using a *B. subtilis* strain that was genetically modified with a fused fragment encoding EDDIE-CAM-W. Up to 159 mg of CAM-W from 1 L of fermentation supernatant was obtained. The size of the purified recombinant CAM-W was consistent with the expected molecular weight of 3.2 kDa. These findings are important for the application of this novel system as a powerful tool for the efficient production of CAM-W.

## Additional Information

**How to cite this article**: Ji, S. *et al*. Efficient biosynthesis of a Cecropin A-melittin mutant in *Bacillus subtilis* WB700. *Sci. Rep.* **7**, 40587; doi: 10.1038/srep40587 (2017).

**Publisher's note:** Springer Nature remains neutral with regard to jurisdictional claims in published maps and institutional affiliations.

## Supplementary Material

Supplementary Figures

## Figures and Tables

**Table 1:** MICs of CAM-W for different bacteria.

Strains	CAM-W MIC (mg/L)	
	Biosynthesized	Chemically synthesized
*E. coli* 15224	0.3 ± 0.18	0.3 ± 0.13
*P. aeruginosa* ATCC 90271	0.6 ± 0.14	0.6 ± 0.17
*S. aureus* ATCC 29213	2.5 ± 0.20	2.6 ± 0.32
*S. pyogenes* ATCC 10389	2.1 ± 0.19	2.2 ± 0.25
*S. sonnei* ATCC 25931	0.8 ± 0.17	0.9 ± 0.22

Each experiment was repeated three times, and the mean values are expressed as the mean ± standard deviation (SD).
