# Corrigendum: Efficient biosynthesis of a Cecropin A-melittin mutant in *Bacillus subtilis* WB700

**DOI:** 10.1038/srep42154

**Published:** 2017-02-09

**Authors:** Shengyue Ji, Weili Li, Abdul Rasheed Baloch, Meng Wang, Hengxin Li, Binyun Cao, Hongfu Zhang

Scientific Reports 7: Article number: 4058710.1038/srep40587; published online: 01 10 2017; updated: 02 09 2017

In the original version of this Article, Hongfu Zhang was incorrectly affiliated with ‘Institute of Animal Science, Chinese Academy of Agricultural Sciences, Beijing 100094, China’.

The correct affiliation is listed below:

State Key Laboratory of Animal Nutrition, Institute of Animal Sciences, Chinese Academy of Agricultural Sciences, Beijing 100094, China.

This error has now been corrected in the PDF and HTML versions of the Article.

