# Corrigendum: Efficient biosynthesis of a Cecropin A-melittin mutant in *Bacillus subtilis* WB700

**DOI:** 10.1038/srep46007

**Published:** 2017-05-26

**Authors:** Shengyue Ji, Weili Li, Abdul Rasheed Baloch, Meng Wang, Hengxin Li, Binyun Cao, Hongfu Zhang

Scientific Reports 7: Article number: 4058710.1038/srep40587; published online: 01 10 2017; updated: 05 26 2017

In the original version of this Article the affiliations were incorrect. The correct affiliations are listed below:

State Key Laboratory of Animal Nutrition, Institute of Animal Sciences, Chinese Academy of Agricultural Sciences, Beijing 100094, China.

Shengyue Ji, Weili Li, Hengxin Li & Hongfu Zhang.

College of Animal Science and Technology, Northwest A&F University, Yangling, Shaanxi 712100, China.

Shengyue Ji, Weili Li & Binyun Cao.

College of Veterinary Medicine, Northwest A&F University, Yangling, Shaanxi 712100, China.

Abdul Rasheed Baloch & Meng Wang.

These errors have now been corrected in the PDF and HTML versions of the Article.

