# Supplementary material for: Efficient biosynthesis of a Cecropin A-melittin mutant in Bacillus subtilis WB700
Source: Sci Rep. 2017 Jan 10;7:40587. doi: 10.1038/srep40587 (PMC5223193; doi:10.1038/srep40587)
Supplement: Supplementary Figures [file srep40587-s1.pdf]

Title: Efficient biosynthesis of a Cecropin A-melittin mutant in *Bacillus subtilis* WB700

Running title: Biosynthesis of cecropin A-melittin mutant.

Authors: Shengyue Ji[a, b], Weili Li[a, b], Abdul Rasheed Baloch[c], Meng Wang[c], Hengxin Li[a], Binyun Cao[b, #], Hongfu Zhang[a, #]

Addresses

[a]Institute of Animal Science, Chinese Academy of Agricultural Sciences, Beijing 100094, China.
[b]College of Animal Science and Technology, Northwest A&F University, Yangling, Shaanxi 712100, China.
[c]College of Veterinary Medicine, Northwest A&F University, Yangling, Shaanxi 712100, China.

[#]To whom correspondence should be addressed: jiziqing2010@163.com; HongfuZhang2016@163.com

Tel.: +86 29 87092102; +86 10 62816249.

Fax: +86 29 87092164; +86 10 62816249.

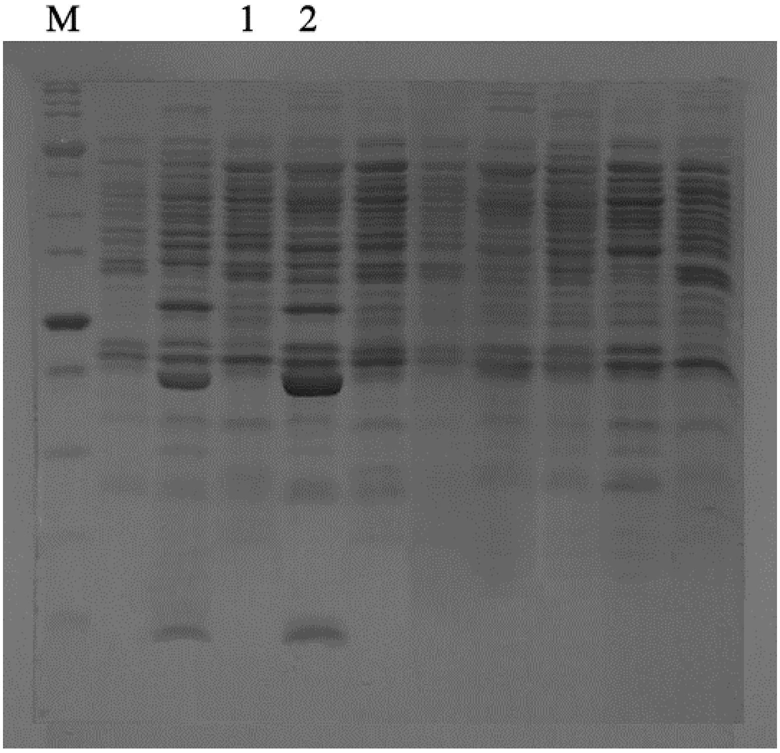

Supplementary figure 1

Supplementary Figure 1. Full-length gel used for tricine-SDS-PAGE analysis of total extracellular proteins.

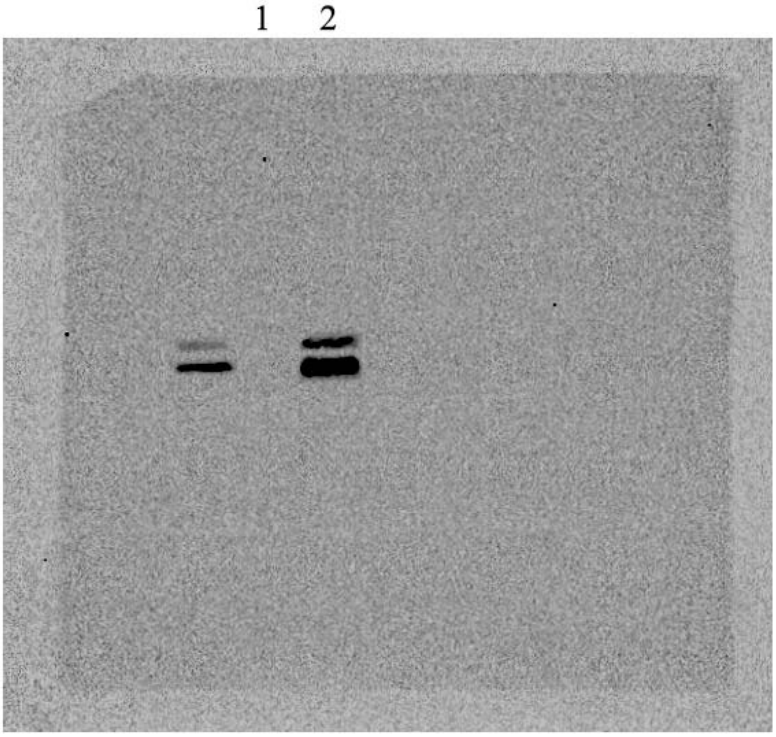

Supplementary figure 2

Supplementary Figure 2. Full-length blot used for western blotting analysis of EDDIE from the total extracellular proteins.

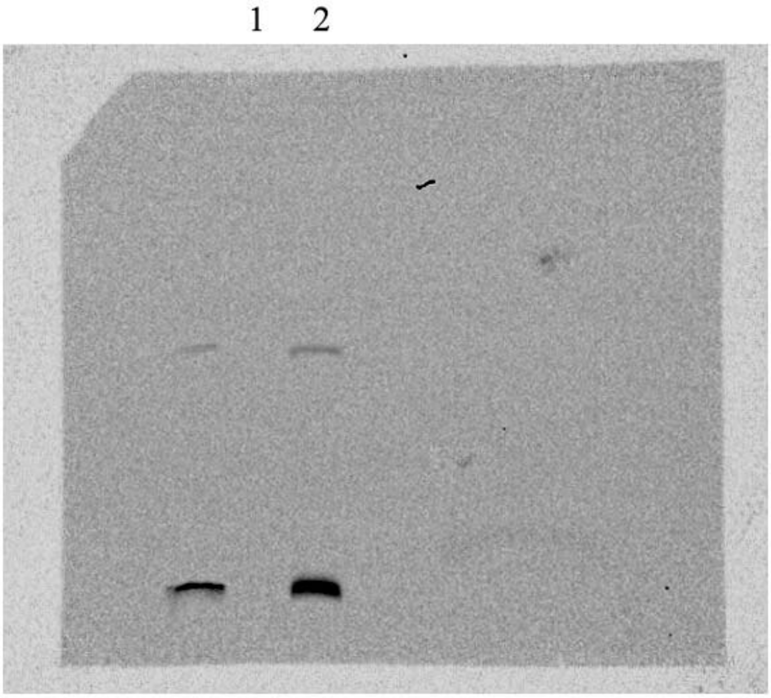

Supplementary figure 3

Supplementary Figure 3. Full-length blot used for western blotting analysis of CAM-W from the total extracellular proteins.
